# Supplementary figures and images for: N-cadherin dynamically regulates pediatric glioma cell migration in complex environments
Source: J Cell Biol. 2024 Mar 13;223(6):e202401057. doi: 10.1083/jcb.202401057 (PMC10937189; doi:10.1083/jcb.202401057)

SourceDataF3

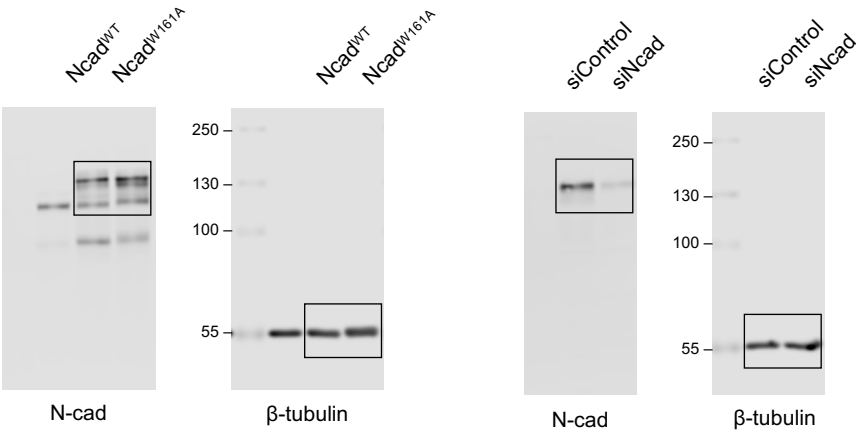

Supplement: SourceData F3 — is the source file for Fig. 3. [file JCB_202401057_SourceDataF3.pdf]

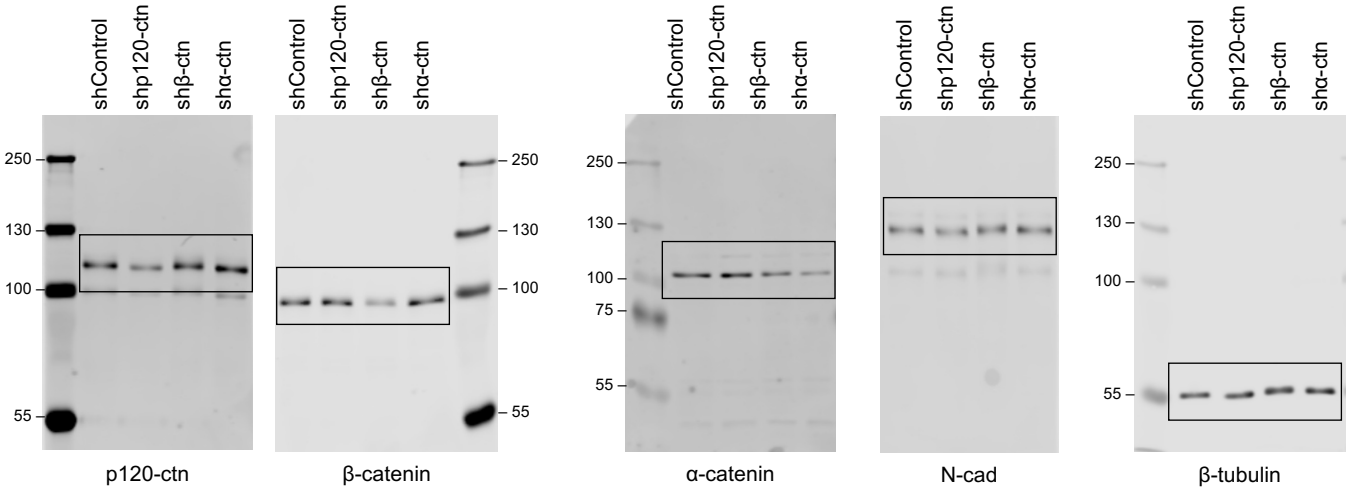

Supplement: SourceData F4 — is the source file for Fig. 4. [file JCB_202401057_SourceDataF4.pdf]

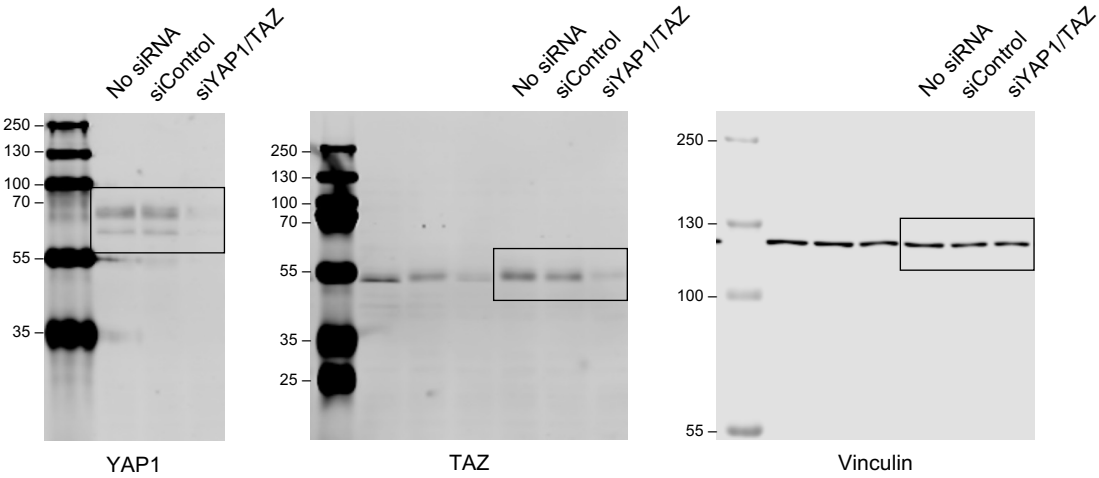

Supplement: SourceData F8 — is the source file for Fig. 8. [file JCB_202401057_SourceDataF8.pdf]

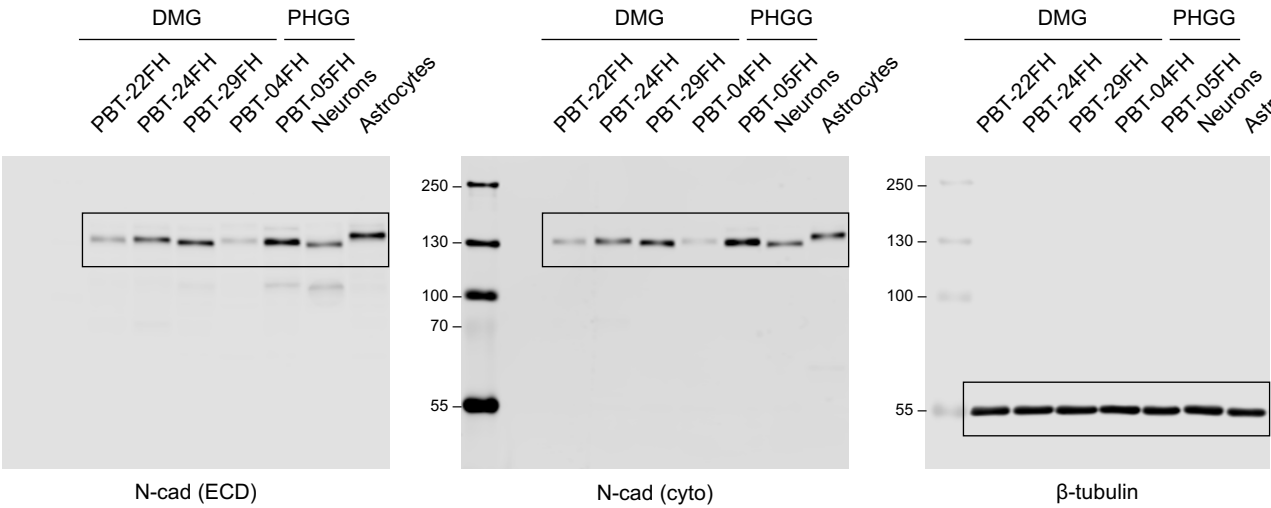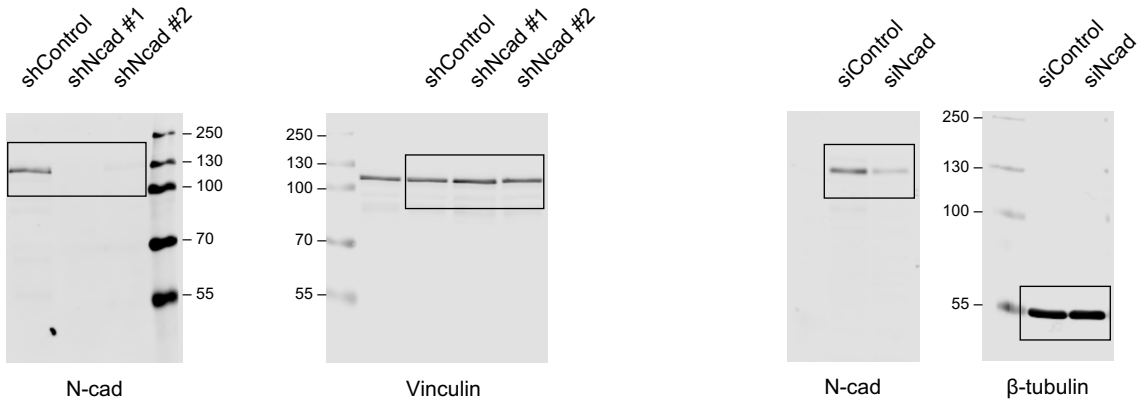

Supplement: SourceData FS1 — is the source file for Fig. S1. [file JCB_202401057_SourceDataFS1.pdf]

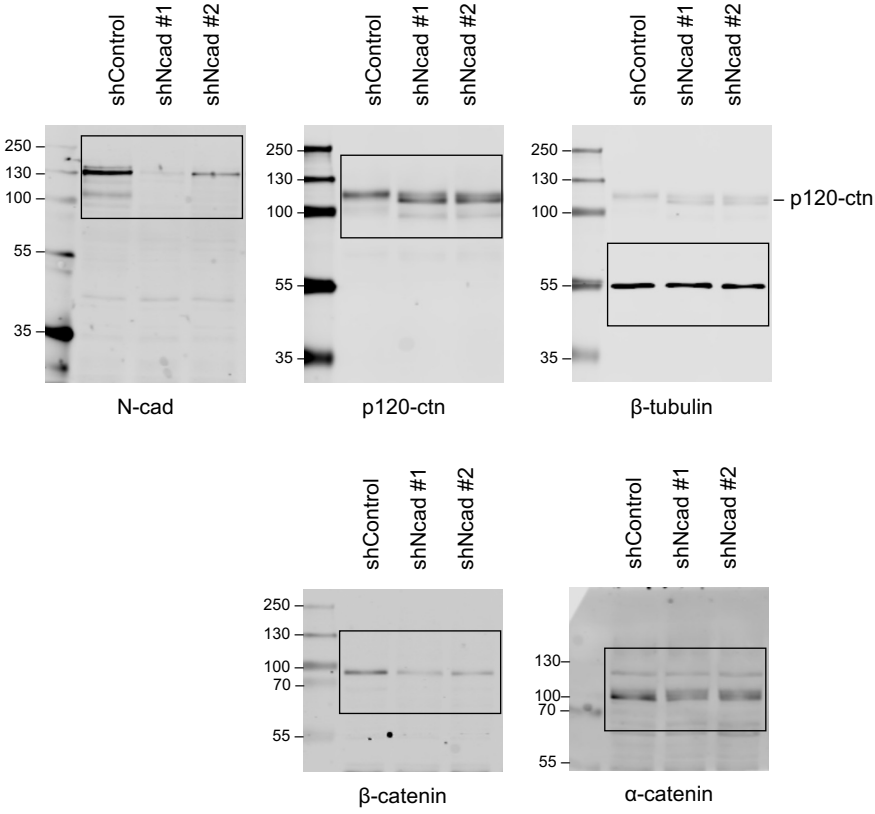

Supplement: SourceData FS2 — is the source file for Fig. S2. [file JCB_202401057_SourceDataFS2.pdf]

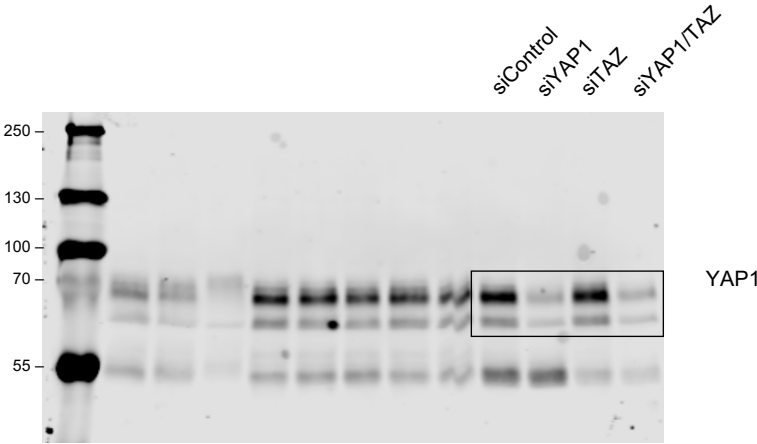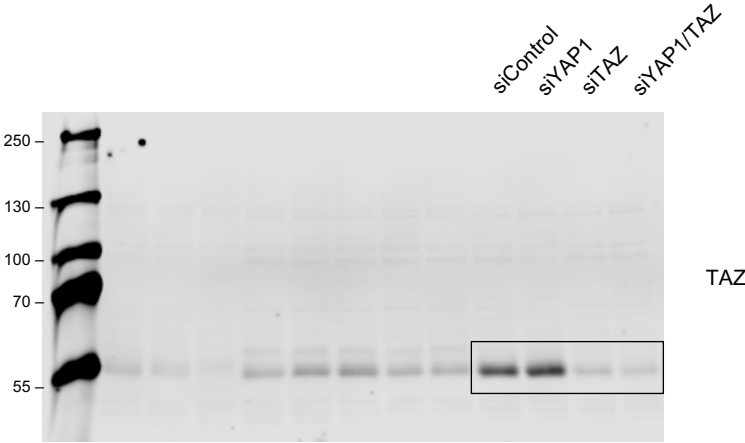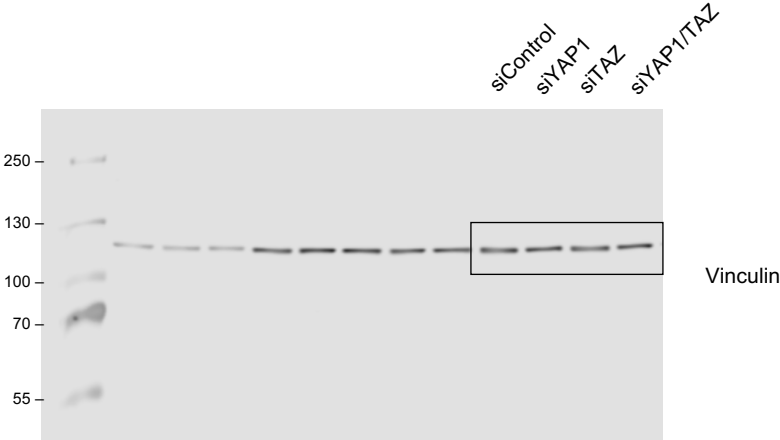

Supplement: SourceData FS5 — is the source file for Fig. S5. [file JCB_202401057_SourceDataFS5.pdf]
